# A Partial Hydatidiform Mole in an Ovarian Ectopic Pregnancy: An Exceptional Occurrence

**DOI:** 10.3390/diagnostics15162024

**Published:** 2025-08-13

**Authors:** Maria Paola Bonasoni, Roberta Zuntini, Khush Shah, Loredana De Marco, Eleonora Zanetti, Luca Pagliai, Immacolata Blasi, Emanuela Carossino, Alice Ferretti, Vincenzo Dario Mandato, Lorenzo Aguzzoli

**Affiliations:** 1 Pathology Unit, Azienda USL-IRCCS di Reggio Emilia, 42122 Reggio Emilia, Italy; loredana.demarco@ausl.re.it (L.D.M.); eleonora.zanetti@ausl.re.it (E.Z.); 2 Clinical Genetics Unit, Azienda USL-IRCCS di Reggio Emilia, 42122 Reggio Emilia, Italy; roberta.zuntini@ausl.re.it (R.Z.); luca.pagliai@ausl.re.it (L.P.); 3 Department of Medicine, Lake Erie College of Osteopathic Medicine at Seton Hill, Greensburg, PA 15601, USA; ksshah0318@gmail.com; 4 Unit of Obstetrics and Gynecologic Oncology, Azienda USL-IRCCS di Reggio Emilia, 42122 Reggio Emilia, Italy; immacolata.blasi@ausl.re.it (I.B.); emanuela.carossino@ausl.re.it (E.C.); alice.ferretti@ausl.re.it (A.F.); vincenzodario.mandato@ausl.re.it (V.D.M.); lorenzo.aguzzoli@ausl.re.it (L.A.)

**Keywords:** ovarian ectopic pregnancy, partial hydatidiform mole, Short Tandem Repeat (STR)

## Abstract

**Background and Clinical Significance**: Ovarian ectopic pregnancy (OEP) is a rare occurrence, and molar degeneration is even more exceptional. Differential diagnosis between a partial and complete hydatidiform mole is paramount as the complete type carries a higher risk of post-molar gestational trophoblastic neoplasia. Herein, we describe a case of a partial mole in an OEP (OPHM) with thorough investigations. **Case Presentation**: A 39-year-old woman presented at 6 weeks of amenorrhea with abdominal pain and vaginal bleeding. Ultrasound showed no intrauterine pregnancy, but an ovarian cyst suspicious for OEP. The patient underwent surgical removal of the cyst. Histological diagnosis was suspicious for OPHM with only one abnormal villous. Immunohistochemistry for p57^kip2^ and fluorescent in situ hybridization (FISH) were not conclusive. STR-based (Short Tandem Repeat) molecular technique demonstrated the chromosomal asset of 69,XXX, confirming the diagnosis of OPHM. The patient was fully monitored for 1 year with periodic measurements of beta-hCG levels. After that period, the patient was in good health and disease-free. **Conclusions**: Histologically, ancillary techniques might not be sufficient to confirm the diagnosis of a hydatidiform mole, especially if the tissue available is scarce. In this case, STR has been demonstrated an effective tool in defining the chromosomal asset, even in paraffin-embedded samples.

## 1. Introduction

An ectopic pregnancy (EP) is defined as the development of the fertilized egg outside the endometrial cavity. This anomaly can occur in about 2% of all pregnancies, but the real incidence may be higher due to scarce reporting. EP may present as a high-risk medical condition, being the most frequent cause of first-trimester mortality, accounting for approximately 4% of all pregnancy-related deaths [1].

Risk factors for EP include a previous EP, fallopian tube damage (like pelvic infections or prior pelvic surgery), smoking, use of an intrauterine device (IUD), infertility, and age above 35 years. However, more than 50% of EPs develop in women without risk factors. Therefore, keeping a high level of clinical suspicion during the initial assessment is fundamental for ensuring early detection [1]. EPs are most commonly located in the fallopian tube, typically in the ampulla, accounting for about 90% of cases. However, in around 1–3% of cases, EPs can occur in the ovary, cervix, and within the abdomen [1]. Ovarian ectopic pregnancy (OEP) is a rare occurrence, accounting for 1–3% of EPs and 0.03–0.09% of all pregnancies [2]. A primary OEP ensues when the gestational sac implants directly into the ovary. A secondary OEP occurs when the egg is fertilized in the fallopian tube and then expelled backward into the ovary, where it embeds within the ovarian stroma [3]. The main risk factor is the use of IUD, but a previous EP, endometriosis, and pelvic inflammatory disease may also be causative. OEP can be due to In Vitro Fertilization (IVF) in 28.5% of procedures, as tubal reflux may facilitate the implantation of the fertilized egg into the ovary [3]. A hydatidiform mole (HM) occurring in an OEP is almost exceptional, with only 14 cases of complete mole described in the literature [4,5,6,7,8,9,10,11,12,13,14,15,16,17].

To the best of our knowledge, an ovarian partial hydatidiform mole (OPHM) has never been documented. Herein, we present a case of an OPHM developed in a follicular cyst/early corpus luteum (CL) with thorough investigations including histology and immunohistochemistry for p57, ploidy demonstration with HER2 fluorescent in situ hybridization (FISH) [18], and STR-based (Short Tandem Repeat) molecular technique for determination of autosomal and sex chromosome number. The case is presented and discussed.

## 2. Case Presentation

### 2.1. Clinical History

**Figure 1:**
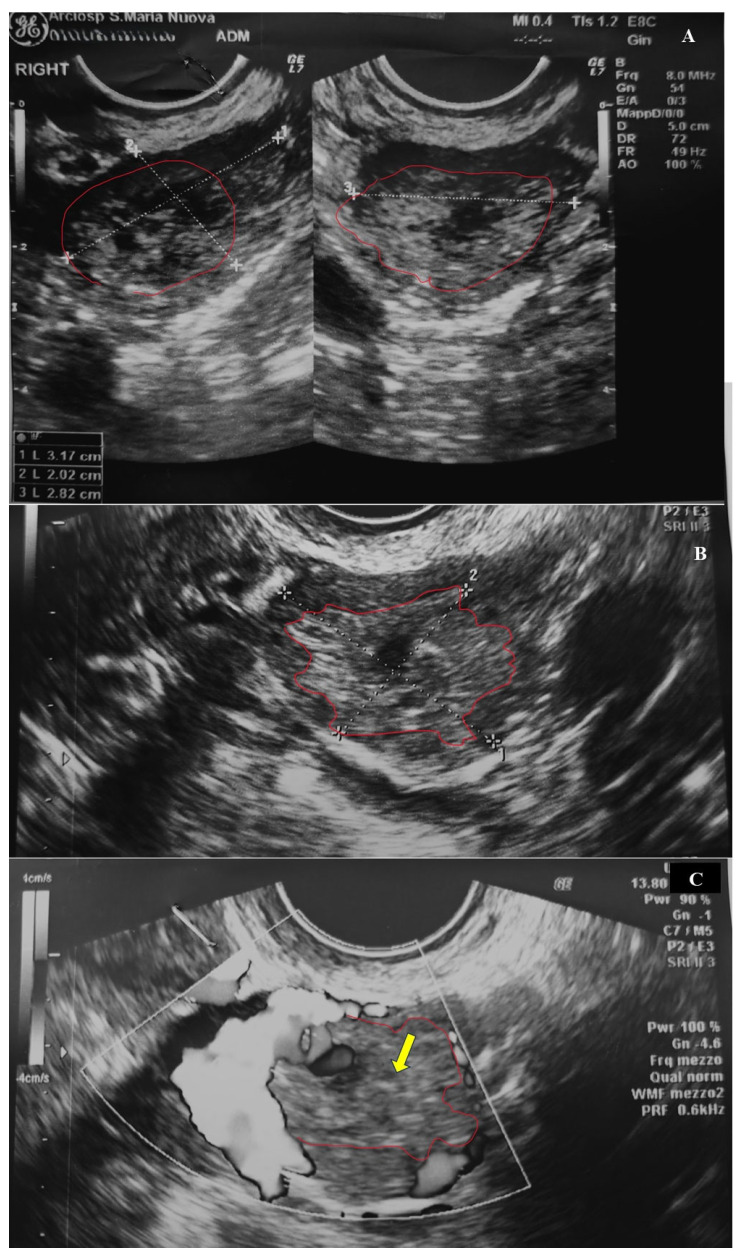
Right ovarian cyst at US: (**A**,**B**) iso-hypoechoic cyst with irregular profile of the ovarian parenchyma (red line). (**C**) Peripheral blood flow associated with a mild Swiss cheese appearance (yellow arrow).

A 39-year-old woman, gravida 2 para 1, presented at 6 weeks + 2 days of amenorrhea to the emergency department complaining of abdominal pain and vaginal bleeding, which had started 3 h prior to the admission. During the gynecological examination with a speculum, bloody brownish traces were observed in the vagina, but there was no active bleeding. Transvaginal sonography (TVS) showed a proliferative endometrial lining measuring 13.5 mm in maximum thickness, but no intrauterine gestational sac. Both fallopian tubes were not dilated. The left ovary presented an anechoic cystic formation of 18 × 10 mm. The right ovary showed a superior round formation of 21 × 20 mm with peripheral vascularization. The content appeared heterogenous with an internal avascular component compatible with blood collection (“Swiss cheese” appearance). Inferiorly, there was the ovarian parenchyma, unremarkable (Figure 1).

No pelvic effusions were detected. A diagnosis of probable right ovarian ectopic pregnancy was made. Beta-hCG levels were 6723 IU/L and 5924 IU/L three and one days before the visit. As the patient was clinically stable, she was discharged with a follow-up US in two days. Subsequent beta-hCG levels were 6703 IU/L and 7189 IU/L after one and two days.

In the following days the beta-hCG levels were 6703 U/L and 7189 U/L, after one and two days, respectively. Two days after the initial visit, TVS still showed no intrauterine gestational sac and the same right ovarian cyst. The patient underwent laparoscopic removal and intrauterine curettage the same day. Both samples were sent for histology. Three days after surgery beta-hCG levels were decreased to 800 U/L.

The patient’s medical history included IVF three years prior with intrauterine embryo implantation. This led to a full-term pregnancy of a healthy male infant approximately two and a half years before the ovarian cyst removal.

### 2.2. Histological Examination

The samples sent for histological examination were the endometrial curettage and the ovarian cyst formation.

The endometrium was hypersecretory with decidualization of the stromal cells. No villous structures were detected. The morphological changes were compatible with early pregnancy, though ectopic.

**Figure 2:**
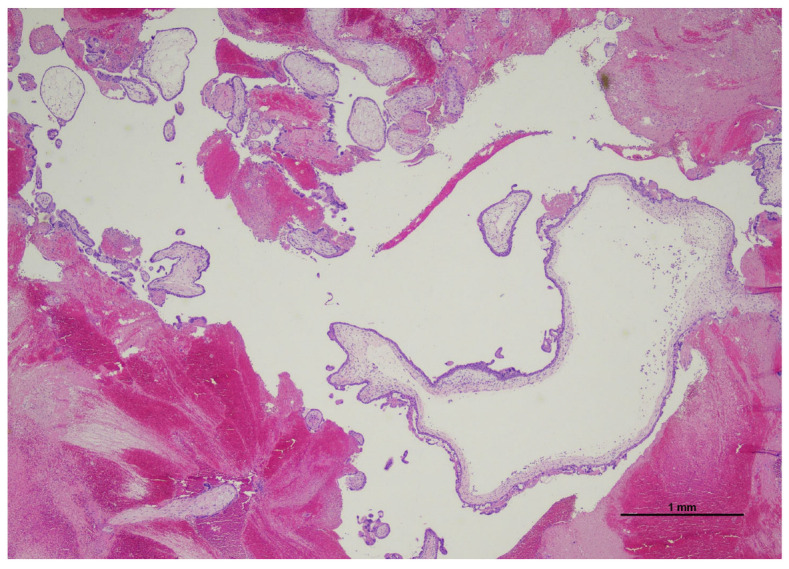
Ovarian ectopic pregnancy: the submitted tissue was composed of villi and partially organized blood. One villous (bottom left) was suspicious for molar degeneration as presented an inner cistern and focal trophoblast proliferation (Hematoxylin and Eosin, 2 HPF).

The ovarian cyst was composed of villous structures, irregularly shaped, with focal trophoblast proliferation and focal hydropic changes. Only one villous showed abnormal inner cistern formation with patchy trophoblast atypical proliferation (Figure 2).

**Figure 3:**
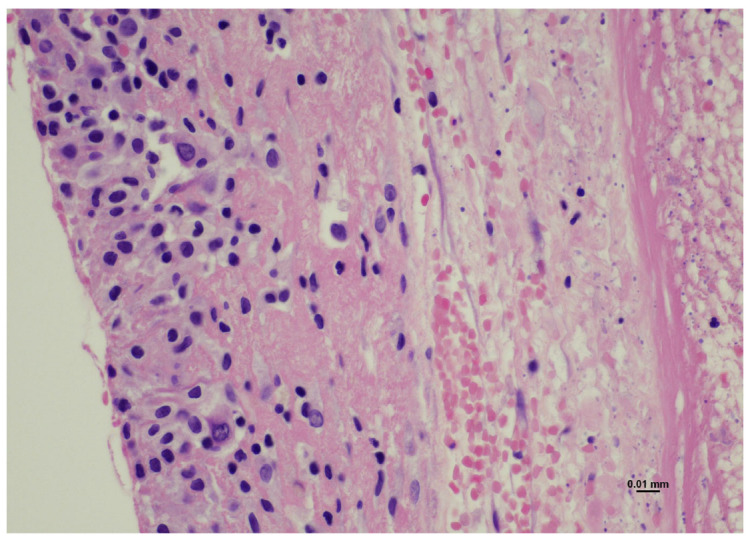
Ovarian ectopic pregnancy: the cyst presented an outer layer made up of eosinophilic cuboidal cells consistent with a follicular cyst/early corpus luteum (Hematoxylin and Eosin, 20 HPF).

Extravillous implantation trophoblast and arterioles were present. No fetal red blood cells or fetal tissues were detected. All these elements were encapsulated and surrounded by a marked eosinophilic cellular layer compatible with a follicular cyst/early corpus luteum (Figure 3).

**Figure 4:**
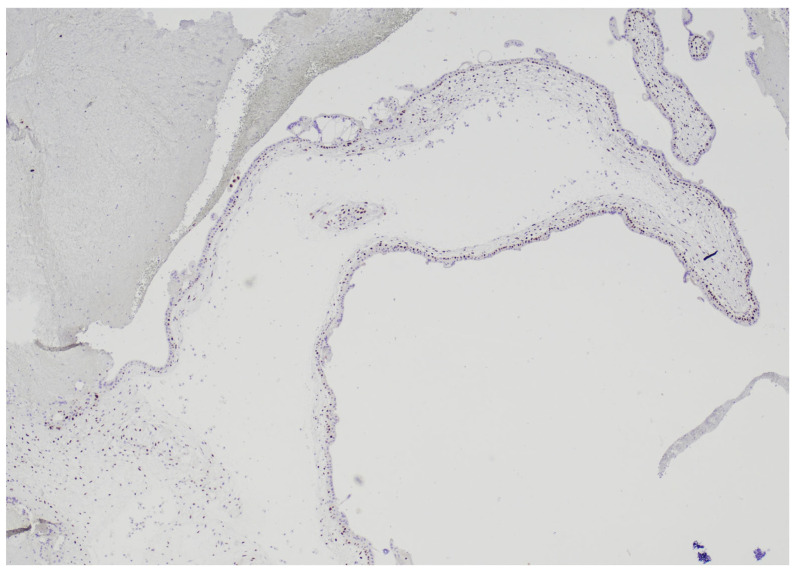
Immunohistochemistry for p57^kip2^: in the suspected hydropic villous the marker was expressed in the cytotrophoblast, arising the suspicion of a partial mole (4 HPF).

Immunohistochemistry for p57^kip2^ showed nuclear positivity in the cytotrophoblast and stromal cells (Figure 4).

These features arose the suspicion of an OPHM. Therefore, HER2 FISH and STR analyses were carried out.

### 2.3. HER2 FISH Analysis

To evaluate ploidy, HER2 FISH was performed according to the manufacturer’s instructions. A 4 μm thick slide from one FFPE block, the one with the suspicious villous, was pretreated with a Vysis IntelliFISH kit (Abbott, Abbott Park, IL, USA) and hybridized with a PathVysion HER-2 DNA probe (Abbott, Abbott Park, IL, USA). Analysis was performed using a Leica DM5500 fluorescent microscope (Leica Microsystem, Milan, Italy) [18]. To determine ploidy, signals referring to the centromere region of chromosome 17 (which is used as reference when evaluating HER2 amplification status) were counted in single, complete, non-overlapping nuclei. In our specific case, on 50 nuclei in interphase only 2 (1%) presented three copies of chromosome 17. As the result was still doubtful, molecular analysis was performed.

### 2.4. STR Analysis

DNA was extracted from 3 sliced sections of 5 μm thickness using a QIAamp DNA FFPE Tissue Kit. In the first step, we used an AmpFLSTR^®^ Identifiler^®^ Plus kit (ThermoFisher Scientific, Waltham, MA, USA) for determining the ploidy of autosomal chromosomes and then a Devyser Compact v3 kit (Devyser Devyser AB, Årsta, Sweden) for determining the number of X chromosomes, according to the manufacturer’s instructions for both. PCR products were run on a 3500 Genetic Analyze (ThermoFisher Scientific, Waltham, MA, USA) and analyzed using GeneMapper v2.0 Software. Table 1 shows the results obtained with the AmpFLSTR^®^ Identifiler^®^ Plus kit, while Table 2 shows the results obtained with Devyser Compact v3 kit.

All STR markers agreed with the presence of a female triploid genomic assortment (69,XXX) (Table 1 and Table 2).

### 2.5. Patient’s Follow-Up

After the histological diagnosis of OPHM, a strict follow-up was planned to detect gestational trophoblastic disease (GTD) with periodic measurements of beta-hCG levels. One month after the surgery, beta-hCG levels were below the threshold (<2 U/L). The test was repeated every 30 days for one year, and the results were always negative.

Thoracic X-rays were performed one month after surgery, and they were normal.

After one year of follow-up, the patient was declared disease-free. After six months, the patient was still in good health and underwent another advanced reproductive technology (ART) procedure with IVF.

## 3. Discussion

EP is estimated to have a global prevalence of approximately 9.69 cases per 100,000 women, based on the latest assessments from 1990 to 2019 [19]. In recent decades, there has been an increase in EPs mostly due to ART. Although early recognition of EP has dramatically changed maternal morbidity and mortality, epidemiologic data mainly comes from high income countries, underestimating the real incidence in middle and low income nations [19]. Tubal EP is the most common location, and OEP is a rare occurrence [1,2].

TVS detection of a ring structure with the yolk sack (+YS), embryo (+EMB), or embryo with heart action (+HA) beyond the uterine cavity was considered as the *specific*, *sure*, or *proving marker* of EP. Recently, the blob and bagel signs have been added. The blob sign refers to an irregular but capsulated well-defined ellipsoidal mass. The bagel sign indicates an echogenic ring of variable thickness encircling a unilocular, round cyst, either centrally or eccentrically located, devoid of content [20]. In EP, urgent intervention depends on the amount and location of abdominal free blood assessed by TVS [21]. A HM can occur in any kind of pregnancy, even ectopic. HMs, complete and partial (CHM, PHM), are part of the gestational trophoblastic disease (GTD), a variety of placental pathologies. HMs are classified as benign placental neoplasms at risk of malignant transformation. GTD also includes the gestational trophoblastic neoplasia (GTN) group, placental tumors exhibiting malignant behavior, including post-molar gestational trophoblastic neoplasia, invasive mole, gestational choriocarcinoma, placental site trophoblastic tumor, and epithelioid trophoblastic tumor [22,23]. HMs result from abnormal gametogenesis or fertilization, leading to placental overexpression of paternally derived genes [22,23].

CHMs are characterized by a chromosomal asset entirely derived from the paternal genome. The most frequent karyotype is 46,XX, resulting from endoreduplication of a single sperm’s haploid genome following fertilization of an enucleated ovum, with complete exclusion of the maternal DNA. Approximately 5–10% of complete moles exhibit a 46,XY karyotype due to dispermic fertilization, which accounts for 10–20% of cases. Fetal tissue, vasculature, and red blood cells are typically absent in complete moles as fetal demise and resorption occurs early, prior to the establishment of the circulatory system [23]. Grossly, the specimen shows vesicular structures, also called “bunch of grapes”, appearing as “snowstorm” at US. Histologically, the villi exhibit a bulbous, “cauliflower-like” appearance with internal cisterns and widespread circumferential trophoblastic hyperplasia involving cytotrophoblast and syncytiotrophoblast. The implantation site and the extravillous trophoblast typically show marked atypia [23].

PHMs typically exhibit a complete triploid chromosomal set composed of two paternal and one maternal haploid genomes. The most common karyotypes are 69,XXX or 69,XXY, arising from fertilization of a haploid ovum either by a single sperm followed by duplication of its genome or, less commonly, by dispermic fertilization. Triploidy with a 69,XYY karyotype is rare, and a 69,YYY karyotype has never been detected. A distinguishing pathological feature of partial moles is the presence of gross or histological evidence of fetal development, such as amniotic tissue or fetal vessels containing red blood cells [22]. Macroscopically, the specimen presents minimal vesicular degeneration. Microscopically, there are two groups of villi, one composed of larger villi with irregular shape and outline and cisterns formation. Circumferential trophoblastic hyperplasia is usually focal, mild to moderate. Implantation site and extravillous trophoblast atypia can be present and focal [23].

Immunohistochemistry for p57^kip2^, can be helpful in distinguishing complete and partial moles. In CHM, nuclear expression of p57^kip2^ is restricted to the intermediate trophoblast and decidual cells, with absence of staining in villous cytotrophoblast nuclei. Conversely, in PHM, p57^kip2^ demonstrates strong nuclear positivity in villous cytotrophoblast, in addition to the previously mentioned sites [23].

This expression is due to the nature of the *p57KIP2* gene, a cyclin-dependent kinase inhibitor located on chromosome 11p15.5, subjected to strong paternal imprinting and expressed exclusively from the maternal allele [24].

Generally speaking, with no reference to a uterine or ectopic pregnancy, differentiating between CHM and PHM is crucial as CHM is associated with a greater risk of maternal complications and a higher likelihood of progressing to GTN. Post-molar GTN develops in approximately 15–20% of CHMs, compared to about 1.5% in PHMs [25].

Molar degeneration in OEP has been described in few cases in the literature, but histology, US, and molecular STR analysis always matched an OCHM [4,5,6,7,8,9,10,11,12,13,14,15,16,17].

In our case, the ovarian cyst presented only one villous suspicious for molar degeneration. Immunohistochemistry for p57^kip2^ showed nuclear positivity in the cytotrophoblast and stromal cells, indicating OPHM. However, FISH analysis was not conclusive as on 50 nuclei in interphase only 2 (1%) presented three copies of chromosome 17. This result might have reflected the coexistence of maternal decidual diploid cells., then STR analysis demonstrated a female triploid genome.

Triploid pregnancies can be classified as digynic, containing two sets of maternal chromosomes and one paternal, or diandric, comprising two paternal sets and one maternal. Karyotype can be 69,XXX; 69,XXY; and 69,XYY [26].

Triploidy can be identified in 1% of conceptions, most of them result in spontaneous abortion in the first trimester with an incidence of almost 20%. Fetal development and survival beyond the first trimester in triploid pregnancies are quite rare, with an estimated occurrence of one triploid fetus per 5000 pregnancies between the 16th and 20th weeks of gestation [26].

In our specific case, in which triploidy 69,XXX was identified, no fetal tissues or nucleated red blood cells were observed. We therefore assumed that the PHM could have been diandric, but the genetic parental contribution was not pursued.

OEP can be classified as intrafollicular (occurring in a corpus luteum) or extrafollicular (embedded in the ovarian stroma) [2]. OEP-intrafollicular type is quite rare, being misdiagnosed as a CL. A CL is usually a unilocular cyst less than 3 cm which can be anechoic or hemorrhagic with a thick, sawtooth wall. Internal echoes due to internal hemorrhage may mimic a solid mass without internal blood flow. Typically, blood flow surrounds the wall of the CL (ring of fire) and may be used to distinguish CL from OEP. The OEP is usually located within the ovarian stroma and cannot be separated from the ovary by pressure from the probe. The presence of a gestational sac or embryo can facilitate the diagnosis [2]. PHM may present with abnormal, heterogeneous, and large placental tissue for gestational age. Compared to CHM, it often presents greater vascularization and embryonic and extraembryonic structures, although altered, and less frequently presents cystic placental tissue (“Swiss cheese”) [27]. In our case, neither CHM nor PHM were suspected, and the ovarian lesion was misdiagnosed with a CL: although the ring of fire was not found and the Swiss cheese modification was not so evident. Moreover, no embryonic and extraembryonic structures were detected. However, it is known that diagnosing a PHM can be more difficult than a complete one, because rigorous diagnostic criteria are often not available [27].

In OEP, symptoms may include abdominal pain and/or vaginal bleeding, often alongside elevated beta-hCG levels.

Maternal morbidity and mortality are associated with OEP rupture, hemoperitoneum, and hemodynamic instability [2]. As soon as the OEP is detected, surgical treatment is mandatory to prevent fatal outcomes.

In our case, the patient presented with vaginal bleeding, but no intrauterine pregnancy was detected at TVS. However, a suspicious cystic formation of 21 × 20 mm was found in the right ovary. The lesion was anomalous as the outer layer was vascularized and the content was compatible with blood. Beta-hCG levels were 7189 U/L at surgery. After the OEP removal, beta-hCG levels progressively decreased. Histological examination was tricky as the tissue available was scarce and only one villous raised the doubt of an OPHM. FISH analysis and immunohistochemistry for p57^kip2^ oriented for an OPHM, but only STR technique identified a triploid chromosomal asset. PHM and CHM have a different prognosis, as in the latter there is a higher occurrence of GTN [25]. Therefore, in ambiguous cases, when histology, immunohistochemistry, and FISH analysis are not diriment, STR can represent a reliable and useful diagnostic tool. Early hydatidiform moles and hydropic abortion can be histologically similar and the usual ancillary tools might not be sufficient to discriminate them. Instead, this technique is highly sensitive and specific and can be used in formalin-fixed, paraffin-embedded tissue, providing the exact chromosomal asset [28].

## 4. Conclusions

OEP is a rare occurrence, and its molar degeneration is almost exceptional. To the best of our knowledge, this was the first case of a PHM in an OEP. The main difficulty of this case was to detect histologically the PHM in the scarcity of the tissue submitted. Ancillary techniques, such as simple morphology, immunohistochemistry, and FISH, might not be sufficient to confirm the diagnosis. STR has been demonstrated an effective tool in defining the chromosomal asset, even in paraffin-embedded tissues.

## Figures and Tables

**Table 1:** In the first column the markers contained in the kit, in the second column their genomic position, in the third column the size range of each allele, in the fourth column the size of the alleles present, and in the fifth column the relevant Combined DNA Index System (CODIS) loci.

Marker	Chromosome Location	Size Range	Allele Size	Allele
D8s1179	8	122–169	123-135-148	8-11-14
D21s11	21q11.2-q21	184–240	206-210	29.2-30.2
D7s820	7q11.21-22	255–292	263-266-279	8-9-12
CSF1PO	5q33.3-34	304–340	314-322-330	9-11-13
D3s1358	3p	111–140	120-128-132-136	14-16-17-18
TH01	11p15.5	162–202	172-181-184	7-9-9.3
D13s317	13q22-31	216–244	232-236-240	12-13-14
D16s539	16q24-qter	252–292	276-279-279	11-12-12
D2s1338	2q35-37.1	306–359	314-318-326	17-18-20
D19s433	19q12-13.1	101–135	116-120	13-14
Vwa	12p12-pter	154–206	173-176-176	16-17-17
TPOX	2p23-2per	221–250	229-229-236	8-8-10
D18s51	18q21.3	261–344	292-292-300	14.2-14.2-21
Amelogenin	X: p22.1-22.3 Y: p11.2	106–111	106	X
D5s818	5q21.31	133–172	149-153-157	11-12-13
FGA	4q28	214–355	244	25

**Table 2:** In the first column the markers contained in the kit, in the second column the size of the alleles present, and in the third column the allele height.

Marker	Allele Size	Allele Height
X1	139-151	850-1659
XY2	191-199	339-723
X3	281-285-269	390-379-360
X9	322-334-338	412-369-358

## Data Availability

The data presented in this study are available on request from the corresponding author.
